# Parameters of central hemodynamics and arterial stiffness as risk markers for dementia: a systematic review

**DOI:** 10.1590/1806-9282.20250114

**Published:** 2025-12-05

**Authors:** Victor Rayan de Freitas Félix, Pedro Pereira Tenório, Matheus Rodrigues Lopes, Romero Henrique de Almeida Barbosa

**Affiliations:** 1 Universidade Federal do Vale do São Francisco – Paulo Afonso (BA), Brazil.

## INTRODUCTION

Hemodynamics refers to the study of blood flow in vessels. Peripheral hemodynamics is the most well-established concept as an etiopathogenic basis for many clinical conditions; however, there has been a shift in scientific thinking, with central hemodynamics, which assesses parameters at the level of the heart and large vessels, including arterial stiffness, becoming increasingly prominent^1^.

Arterial stiffness refers to the abnormal hardening of the vessel wall. It is clinically assessed using central hemodynamic parameters, such as central blood pressure (CBP), pulse wave velocity (PWV), and augmentation index (AIx). These well-established markers of central hemodynamics and arterial stiffness offer valuable insights into arterial health vitality^2-4^.

Scientific evidence suggests that arterial stiffening leads to changes in the brain's small blood vessels through repeated microinfarctions, which, over time, can contribute to the development of dementia. However, there is no consensus on how this process occurs, particularly regarding its clinical relevance and application parameters^4-9^.

Dementia, or major neurocognitive disorder (a term adopted by the Diagnostic and Statistical Manual of Mental Disorders—DSM-V), is characterized as a degenerative process of brain cells that results in a gradual and widespread decline in cognitive functions, mainly affecting memory. It is also recognized as a significant public health problem because it directly impacts society's structure. Dementia causes suffering for the patient and their family members, primarily due to increasing inability and dependence on others to manage daily activities^5-9^.

Among the dementias, it is important to highlight Alzheimer's disease, characterized by changes in the folding of beta-amyloid peptides and the aggregation of tau proteins. It is currently considered the primary cause of degenerative dementia. Additionally, vascular dementia is recognized as the final stage of vascular cognitive impairment, making it the second most common cause of dementia and the leading cause of non-degenerative dementia^10-13^.

Therefore, this study aims to conduct a systematic review of the literature to establish the relationship between parameters of central hemodynamics, arterial stiffness, and dementia, with a primary focus on how these indices can serve as risk markers for dementia.

## METHODS

This systematic review has not been registered in a review registration database; however, it follows the recommendations of the PRISMA Statement^14^, whose objective is to answer the following question: "*Are the parameters of central hemodynamics and arterial stiffness useful as risk markers for dementia?*"

The research question was developed using the PICOS strategy:

- Population: Patients at risk of dementia.
- Intervention: Measurement of central hemodynamic and arterial stiffness parameters using CBP, PWV, and AIx.
- Comparison: Peripheral blood pressure measurement as a risk factor for dementia.
- Outcomes: Applicability of CBP, PWV, and AIx as early risk markers for dementia
- Study design: Original articles and meta-analyses.

### Eligibility criteria

Only original articles and meta-analyses published between 2017 and 2024 on the state of the art about central hemodynamic and arterial stiffness parameters associated with dementia were included. The search for references was limited to studies published in Portuguese and English.

Studies were selected that analyzed outcomes related to the presence or risk of developing dementia and used arterial stiffness parameters to assess these characteristics.

### Exclusion criteria

Studies that did not involve human subjects, studies in which the patients involved did not present a risk of dementia or cognitive impairment, articles that did not analyze the outcomes of interest in this study, studies of low methodological quality, unpublished manuscripts, and conference abstracts were considered ineligible.

### Search strategy and study selection

A bibliographic survey was carried out using the MEDLINE^®^ databases via PubMed^®^ and SciELO from 2017 to 2024. The descriptors used in English are listed in the Medical Subject Headings Terms (MeSH) of the US National Library of Medicine (NLM), and those in Portuguese are listed in the Health Sciences Descriptors (DeCS) of the Virtual Health Library (BVS). These descriptors were used individually or together, using Boolean operators (AND and OR). The search strategy used for PubMed^®^ and SciELO, as well as the descriptors used, can be seen in Table 1.

**Table 1:** Search strategy used in PubMed^®^ and SciELO.

#1	((((((((Arterial Stiffness [MeSH Terms]) OR (Vascular Stiffness)) OR (Stiffness, Vascular)) OR (Vascular Stiffnesses)) OR (Arterial Stiffnesses)) OR (Stiffness, Arterial)) OR (Aortic Stiffness)) OR (Aortic Stiffnesses)) OR (Stiffness, Aortic)
#2	((((((((((((((Dementias, Vascular[MeSH Terms]) OR (Vascular Dementias)) OR (Vascular Dementia)) OR (Subcortical Vascular Dementia)) OR (Dementia, Subcortical Vascular)) OR (Dementias, Subcortical Vascular)) OR (Subcortical Vascular Dementias)) OR (Vascular Dementia, Subcortical)) OR (Vascular Dementias, Subcortical)) OR (Arteriosclerotic Dementia)) OR (Arteriosclerotic Dementias)) OR (Dementia, Arteriosclerotic)) OR (Dementias, Arteriosclerotic)) OR (Binswanger Disease)) OR (Disease, Binswanger)
#3	(#1 AND #2)

### Data extraction and quality analysis

Data analysis was conducted descriptively, with data independently extracted by reviewers using a data extraction table. The collected information for synthesizing the relevant articles included the year of study, location, target audience, sample size, study type, method used to measure arterial stiffness, and the corresponding results for the assessment of dementia.

The methodological quality of the articles was assessed using the National Institutes of Health (NIH) Quality Assessment of Systematic Reviews and Meta-Analyses to determine their overall quality. Possible selection and publication biases cannot be ruled out due to limitations in the search strategy, as the final sample mainly included studies with positive results, and the search was restricted to only two databases and publications in English and Portuguese. Moreover, measurement protocols varied across studies; this variation may introduce measurement error or inconsistency bias.

## RESULTS

Initially, a bibliographic survey was conducted using all the descriptors in the PubMed^®^ database, which yielded 56 studies. Of these, 31 articles were excluded because they either had titles unrelated to the topic or were duplicates. The same search method was applied to the SciELO database, resulting in two articles. After this stage, 27 articles were selected.

**Figure 1:**
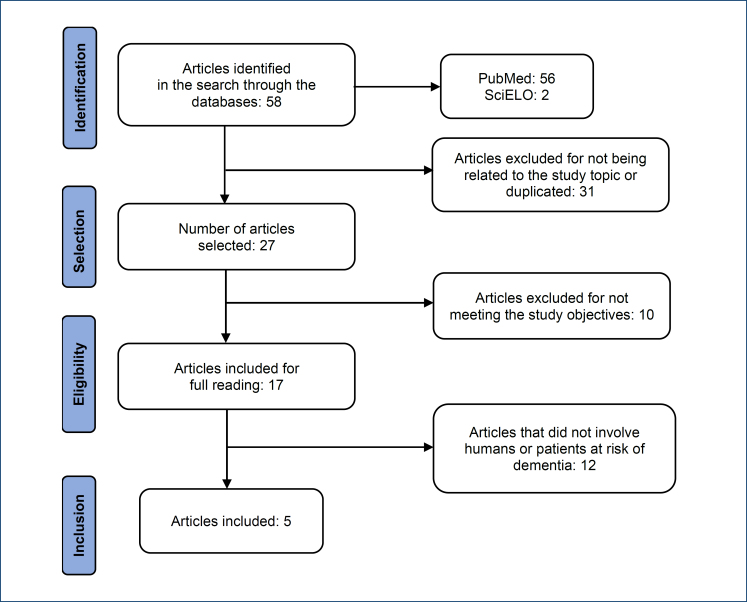
Flowchart of article search for systematic review.

Upon reviewing the titles and abstracts of these studies, 10 articles were excluded because they were not original research or meta-analyses from 2017 to 2024, or they were not written in English or Portuguese. The remaining 17 articles were read in full, and 12 were excluded because they did not involve human subjects or the patients did not have dementia or cognitive impairment. This process resulted in a final sample of five articles, as shown in the flowchart in Figure 1.

Three original articles were included: one observational cross-sectional study, one prospective longitudinal study, one randomized controlled clinical trial, and two meta-analyses. Of these, three studies involved adults in general, while two focused specifically on elderly individuals. The total number of patients evaluated was 64,374. One study focused on patients diagnosed with systemic arterial hypertension, another examined elderly individuals involved in two different physical activity programs, and a third used patients with confirmed dementia. The two meta-analyses included data from both the general population and specific groups of populations^15-19^.

The measurements of central hemodynamics used were PWV in all five studies, with variation in the type of PWV used, such as carotid–femoral pulse wave velocity (PWVcf), brachial–ankle pulse wave velocity (PWVba), aortic pulse wave velocity (PWVao), and ankle–femoral pulse wave velocity (PWVaf); AIx in two studies; and CBP in two studies. The methods for measuring these parameters, such as position, patient condition, and measurement count, were generally consistent; however, different devices were employed in various studies. The most used devices included Complior, SphygmoCor, Omron-Colin, and others such as Mobil-O-Graph^15-19^.

The main characteristics of the articles included are described in Table 2.

**Table 2:** Main data from the studies included in the review.

Author, year, and country	Type of study	Target audience	Sample size	Method used to measure arterial stiffness	Main conclusions
Alvarez-Bueno et al.^15^, 2020, United States	Meta-analysis	Adults	38 studies 43,115 participants	Survey of studies that used PWVcf, PWVba, and PWVao	Negative association between arterial stiffness, measured by PWV, and cognition, specifically executive function, memory, and global cognition. Assessment of PWV may be a useful tool to identify individuals at high risk of cognitive decline or early stages of cognitive decline, to implement interventions aimed at delaying progression to dementia.
Liu et al.^16^,2021, United States	Meta-analysis	Adults	11 studies 20,934 participants	Survey of studies that used PWVcf and PWVao	Aortic stiffness is inversely associated with cognitive function, making it an independent predictor of cognitive impairment and a potential risk factor for dementia, especially in the elderly.
Muela et al.^17^, 2017, Brazil	Cross-sectional observational	Hypertensive adults	211 patients	PWVcf was obtained using the Complior device, when measuring the common carotid artery and femoral artery with the patient in the supine position. The measurements were repeated in 10 different cardiac cycles	Arterial stiffness in patients with hypertension is related to dementia, according to the increase in pressure levels.
Okamoto et al.^18^, 2018, Japan	Randomized controlled clinical trial	Elderly people undergoing physical exercise interventions	68 patients	PWVcf and PWVaf were obtained for 30 s using arterial applanation tonometry, with the Omron-Colin device, before and after the interventions	Both interval walking training and normal walking training were effective in improving cognitive function and reducing arterial stiffness, so that interval walking training was more significant.
Santos et al.^19^, 2017, Brazil	Prospective cohort observational	Elderly people between 60 and 80 years old	46 patients	PWV, AIx, and CBP by taking three measurements on the upper limb using the Mobil-O-Graph device	No significant differences were observed between groups for hemodynamic variables.

AIx: augmentation index; PWV: pulse wave velocity; PWVao: aortic pulse wave velocity; PWVba: brachial–ankle pulse wave velocity; PWVcf: carotid–femoral pulse wave velocity; PWVaf: ankle–femoral pulse wave velocity.

### Pulse wave velocity

PWV is considered a useful parameter to indicate changes related to the arterial bed^15-18^. According to Muela et al., this relationship can be extended by correlating PWV with hypertension and cognitive function, since these authors found increased PWV values with higher blood pressure levels, and this connection suggests a greater risk for cognitive decline and dementia (p<0.05)^17^.

Liu et al. reported that PWVcf measurements ranged from 4.96 to 14.3 m/s on average. They also found that higher PWVao values are associated with an increased risk of cognitive impairment (OR 1.44; 95%CI 1.124–1.845) and dementia (OR 2.1; 95%CI 1.159–3.804) compared to lower PWVao levels. The study further indicates that each 1 m/s rise in PWVao corresponds to a 44% increase in dementia risk, with the risk increasing by 3.9% (OR 1.039; 95%CI 1.005–1.073) for every additional meter per second of PWVao^16^.

Alvarez-Bueno et al. reported PWVcf values between 4.9 and 6.9 m/s, and PWVba values between 15.3 and 23.7 m/s. They categorized the risk of dementia based on cognitive impairment, assessed across three domains: global cognition, executive function, and memory. They found a longitudinal association between PWV and these cognitive domains: −0.21 (95%CI −0.36 to −0.06) for global cognition, −0.12 (95%CI −0.22 to −0.02) for executive function, and −0.05 (95%CI −0.12 to 0.03) for memory. These findings corroborate an inverse correlation between arterial stiffness and each cognitive domain that tends to be affected, which might mean a higher potential risk for dementia. An additional pertinent observation is the early onset of cognitive impairment, which is associated with a higher propensity for such decline in dementia^15^.

Okamoto et al. demonstrated an indirect connection between PWV and cognitive function, concluding that both normal walking training and interval walking training enhance cognitive function and reduce the risk of dementia. Additionally, both methods decreased PWVcf and PWVaf, with IWT more effectively lowering PWV values, especially PWVcf (p<0.05)^18^.

In contrast to the results of previous studies, in the prospective observational cohort study, Santos et al. found that the evaluated PWV values were not different in individuals with vascular dementia, Alzheimer's dementia, or mild cognitive impairment (p=0.26), so the results do not support the use of these methods as aids in differentiating between the two diseases^19^.

### Augmentation index

According to Muela et al. AIx for normotension compared to stage 1 and stage 2 hypertension (p=0.001) serves as a risk marker for cognitive decline and dementia in patients with hypertension, with higher blood pressure levels indicating greater risk. These authors also reached the same conclusion when using AIx75 (augmentation index corrected for a heart rate of 75 bpm), as there was a difference between stage 2 hypertension compared to normotension and stage 1 hypertension (p=0.04)^17^.

However, results found by Santos et al. indicated that AIx values did not vary significantly among individuals with vascular dementia, Alzheimer's dementia, or mild cognitive impairment (p=0.25). The researchers therefore concluded that the data do not support clinical evidence for the use of this parameter in the diagnosis and prevention of dementia^19^.

### Central blood pressure

According to Muela et al., there was an association between CBP (measured as central systolic pressure and central diastolic pressure), systemic arterial hypertension, and cognitive function, such that higher pressure values are related to elevated CBP and increased risk of dementia (p<0.001). Therefore, this marker was likely to be used as a risk predictor for cognitive impairment dementia^17^.

Still, Santos et al. found the opposite result, as the CBP values did not vary significantly when comparing individuals with vascular dementia, Alzheimer's dementia, or mild cognitive impairment (p=0.85 for central systolic pressure and p=0.45 for central diastolic pressure), which rules out the possibility of using this marker for clinical diagnosis method^19^.

## DISCUSSION

The pathophysiological basis explains the link between arterial stiffness parameters and dementia revolves around the so-called cardiovascular aging continuum, which suggests that as vascular injury progresses, vital organs with smaller blood vessels tend to sustain more structural damage. For example, the brain develops clinically insignificant cerebral microinfarcts that, over time, may lead to cognitive deficits and dementia^20^.

Few studies aim to clarify this relationship, especially regarding clinical data. The main parameters analyzed are closely linked to the development of dementia, particularly those of vascular origin^1^. It is believed that these parameters can enable early detection of the potential risk for dementia overall, assisting in the identification of individuals at risk of developing dementia or experiencing milder conditions at an early stage, thereby facilitating immediate preventive measures. This early identification is particularly significant in clinical practice, as numerous studies have observed an inverse correlation between arterial stiffness and cognitive function, indicating that elevated values of arterial stiffness parameters may serve as warning indicators for initial cognitive decline.

PWV (including PWVao, PWVba, PWVcf, and PWVaf) has been demonstrated to be the most reproducible and efficacious parameter owing to its ease of measurement, cost-efficiency, and the robust correlation with alterations pertinent to the arterial bed, particularly when utilizing PWVcf. There exists no consensus within the literature regarding the precise value of PWV, either for establishing normative ranges or for evaluating dementia risk. Nonetheless, PWVcf has been identified as the most promising biomarker for this purpose and recommended as a valuable adjunctive tool for risk stratification in hypertensive patients, where the relationship between arterial stiffness and cognitive decline is more pronounced^15-18^.

It was found that there is limited data on the use of AIx as a marker of dementia, and these data are even contradictory, indicating the need for further research on its analysis. Consequently, it can be inferred that the application of AIx for this purpose is more pertinent in specific populations, such as hypertensive patients, and its use as a diagnostic tool for dementia in general is feasible; however, its effectiveness for the differential diagnosis of various types of dementia is constrained. This limitation also pertains to its adjusted value for heart rate, AIx75. This constraint is evident in clinical practice, where AIx, although potentially beneficial in subgroups, lacks the necessary sensitivity and specificity to effectively distinguish between different types of dementia, such as vascular dementia and Alzheimer's disease^17,19^.

Consistent with the evidence found for AIx, CBP, a parameter of central hemodynamics that has been minimally studied in relation to cognitive impairment and dementia, is assessed. Additionally, it is suggested that it is more suitable for use in patients from more specific populations, particularly those in advanced stages of hypertension, and that it has limited validation as a differential diagnostic tool marker^17,19^.

Overall, four studies indicate that these arterial stiffness parameters are good predictors of risk for dementia, and one study found the opposite result, which rules out the possibility of using these parameters clinically for the diagnosis and prevention of dementia. However, it is worth noting that this negative association may occur due to external influences, since these indices are highly affected by various medications such as antihypertensive vasodilators frequently used by the elderly with cognitive impairment and by the practice or lack of physical activity^15-19^.

In this context, it is also important to acknowledge that lifestyle interventions, such as regular physical activity, have demonstrated a beneficial effect on arterial stiffness. Studies indicate that walking programs (both continuous and interval) can not only reduce PWV but also improve cognitive function, thereby emphasizing the significance of these strategies as preventive measures integrated into clinical practice care^15-19^.

This study acknowledges certain limitations that should be carefully considered when interpreting the results. The primary limitation involves the small number of studies included in the final sample, combined with the substandard methodological quality of some of these studies, mainly due to the predominance of observational designs and relatively small sample sizes. These factors diminish the strength of the conclusions and restrict the applicability of the findings to diverse populations. Arterial stiffness, as a physiological measure, is influenced by various transient factors; the lack of consistent control over these factors in the included studies significantly impacts the external validity of the results. Furthermore, only a limited number of studies provided explicit cutoff values for the parameters examined, thereby complicating standardized clinical application as risk markers for dementia. The substantial methodological variability across the studies (including differences in the devices utilized, types of PWV analyzed, and measurement protocols) also complicates the comparison of findings and the establishment of standardized values for reliable clinical use. Additionally, although some studies demonstrated a correlation between these parameters and cognitive function, establishing causality or assessing the utility of such markers in distinguishing different types of dementia remains infeasible. Therefore, arterial stiffness parameters should currently be regarded as nonspecific risk markers: indicators of increased vascular risk but not substitutes for specific diagnostic procedures or methods.

## CONCLUSION

The arterial stiffness parameters most closely associated with the overall development of dementia include PWV, AIx, and CBP, with PWVcf being the most extensively studied parameter. An inverse correlation was identified between measures of arterial stiffness and cognitive function, indicating that elevated levels of these markers are linked to diminished cognitive performance, thereby increasing the risk of dementia onset. The data imply a potential clinical benefit in employing these parameters as predictive indicators of dementia, particularly among hypertensive patients, although they do not facilitate differentiation between various dementia types. Additionally, a correlation was observed between physical exercise and reductions in arterial stiffness and the associated risk of dementia.

However, it is necessary to conduct new studies with a higher level of evidence, such as double-blind randomized controlled trials, aimed at defining and standardizing cutoff values capable of predicting the risk of dementia.

## Data Availability

The datasets generated and/or analyzed during the current study are available from the corresponding author upon reasonable request.
